# The Osteogenesis Mechanisms of Dental Alveolar Bone Socket Post Induction with *Hydroxyapatite* Bovine Tooth Graft: An Animal Experimental in *Rattus norvegicus Strain Wistar*

**DOI:** 10.1055/s-0042-1756691

**Published:** 2022-10-28

**Authors:** Nanik Zubaidah, Dian Dwi Pratiwi, Maria Margaretha S. Nogo Masa, Ernie Maduratna Setiawatie, Sri Kunarti

**Affiliations:** 1 Doctoral Program of Medical Science, Faculty of Medicine, Universitas Airlangga, Indonesia; 2 Department of Conservative Dentistry, Faculty of Dental Medicine, Universitas Airlangga, Surabaya, Indonesia; 3 Post Graduate Program of Conservative Dentistry Specialist, Faculty of Dental Medicine, Universitas Airlangga, Surabaya, Indonesia; 4 Department of Periodontics, Faculty of Dental Medicine, Universitas Airlangga, Surabaya, Indonesia

**Keywords:** *hydroxyapatite* bovine tooth graft, osteogenesis, osteoblast, osteoclast, dental health

## Abstract

**Objectives** Surgical endodontics (hemisection) commonly involves the alveolar bone socket and the periradicular tissue. In today's era, optimizing the bone healing process is updated by using bone graft induction. This study explores the mechanisms of bone healing of the alveolar bone socket post-dental extraction of Wistar rats after administration of a bovine tooth graft (hydroxyapatite bovine tooth graft [ *HAp* -BTG]).

**Materials and Methods** Fifty Wistar rats were randomly selected into two groups, control and treatment, and into five subgroups on days 3, 7, 14, 21, and 28. The postextraction socket was filled with polyethylene glycol (PEG) as the control and PEG + *HAp* -BTG as the treatment group. On days 3, 7, 14, 21, and 28, Wistar rats were sacrificed, mandibles were taken, paraffin blocks were made, cut 4 µm thick, and made into glass preparations for microscopic examination. The variable analysis was performed by staining hematoxylin-eosin for osteoblasts (OBs) and osteoclasts (OCs) and immunohistochemistry for runt-related transcription factor 2 (RUNX2), osterix (OSX), osteocalcin (OCN), bone morphogenic protein (BMP) 2. We analyzed the expressed cell count per microscope field.

**Results** In general, the number of cell expressions in the treatment group was significantly higher and faster, except for significantly lower OC. The high variables peak occurred on day 14 for RUNX2 and OCN, on day 7 for OSX, while OB significantly increased on day 21 and remained until day 28. The decrease of OC cells occurred on day 7 and remained low until 28 days. BMP2 was first dominantly induced by *HAp* -BTG, then the others.

**Conclusion** *HAp* -BTG can induce higher and faster bone healing biomarkers. BMP2 is the dominant first impacted. On the 28th day, it did not significantly express the suppression of OC by OB, which entered the bone formation and remodeling step.

## Introduction

Endodontic surgery is a part of the field of endodontics through hemisection procedures. This procedure involves the alveolar bone socket, which will impact the defect in the alveolar bone and the periradicular tissue of the surrounding teeth. Trauma that occurs due to tooth extraction will experience a natural healing process by going through three stages of wound healing: inflammation, proliferation, and remodeling. In the bone healing processes, there is the involvement of osteoblasts (OBs) and osteoclasts (OCs). 1 2

The reconstruction of bone defects is still a challenge for endodontists in the field of endodontic surgery. It is because the healing process is often interrupted or even fails. Ideally, the success of treatment depends on new bone regeneration. In the clinical practice for improving the bone healing process, commonly a substitute material, namely bone graft, is widely used in regenerative bone procedures. 3 4 5 Classification of grafting materials includes autograft, allograft, alloplastic graft, and xenograft. The xenogenic graft material, bovine hydroxyapatite ( *HAp* ), is commonly used in dentistry. This graft has osteoconductive and osteoinductive properties. It allows new bone tissue to grow in the spaces between its mineral particles. 6 7

Xenografts are commonly used as an alternative to fillers scaffolds. They are relatively easy to use in the maintenance of dental alveolar bone sockets and facilitate bone formation and promote wound healing. Many current studies have developed *HAp* as a bone graft material. 8 9 Still, so far, there has been limited research on the use of *HAp* bovine tooth graft ( *HAp* -BTG), a graft material derived from bovine teeth.

Bovine teeth have inorganic and organic components that resemble human teeth components. 10 The organic ingredients of dentin and cementum include type I collagen and various growth factors such as bone morphogenic proteins (BMPs). Type I collagen occupies approximately 90% of the tissue's organic content, and the rest is a noncollagenous protein (NCP), biopolymers, citrate, lactate, lipids, and others. NCPs are a specific NCP in dentin. It includes osteopontin (OPN), osteocalcin (OCN), bone sialoprotein (BSP), and osterix (OSX). The others NCPs are runt-related transcription factor 2 (RUNX2) and dentin phosphoprotein. 11 12

BMP has an essential role in embryonic development, including brain and bone formation. BMP-2 increases OCN expression, and its short-term expression is required to sufficiently induce bone formation. BMP-2 also has a unique role in postnatal bone formation. 13 The expression of the OCN gene increased the expression of OSX and RUNX2 as a typical marker of OB function. Different from RUNX2, OCN is a marker of end-stage differentiation. 14 RUNX2, as a pre-OB, is a transcription factor closely related to the OB phenotype. OSX is a gene transcription factor identified at the end of the differentiation of pre-OB cells to OB cells. OSX regulates late-stage osteogenesis and inhibits chondrogenesis. 15

Many studies have succeeded in using bone graft material from bovine bone containing microsized *HAp* . Limited information is available on the study of *HAp* -BTG material, especially for the osteogenesis process of alveolar bone sockets. The bovine bone xenograft study showed the significant expression of RUNX2, type I collagen, alkaline phosphatase (ALP), and OCN within 14 and 28 days. 16 Most studies did not include more sequences on time frames and also limited any information about the conducing of time and biomarkers in the process of bone healing. When (during the time frame) and what biomarkers are expressed in the bone healing process, especially bovine material (bone or teeth), also need to be expanded.

This study will explore the cellular and subcellular mechanisms of bone healing by applying *HAp* -BTG graft material in dental socket postdental extraction in Wistar rat. The core factors were used as indicators, namely BMP-2, RUNX2, OSX, OCN, OBs, and OCs. This study hypothesized that most biomarkers would increase faster healing in the treatment group rather than control.

How about the process and mechanisms of bone healing based on the interaction of biomarkers after induction with HAp-BTG? The maximum expression of factors for bone regeneration after applying *HAp* -BTG also needs to be explored.

## Materials and Methods

This research is an experimental study using laboratory animal using a posttest-only control group design. This study got ethical approval from the Ethics Committee, Faculty of Dental Medicine, Universitas Airlangga, Surabaya, Indonesia (348/HRECC.FODM/VII/2020). We conducted the study at the Biochemistry Laboratory, Faculty of Medicine, Universitas Airlangga, Surabaya, Indonesia.

The experimental animals were *Rattus norvegicus strain Wistar* aged 12 to 14 weeks, male with a body weight of 250 to 300 g, healthy rats, no tooth decay, or defects in the whole body. 16 17 The material used is *HAp* -BTG powder type (particle size is ∼3.5 µm) which was sterilized by gamma rays at BATAN (National Atomic Energy Agency = National Atomic Energy Center), Jakarta, Indonesia. The preparation of *HAp* -BTG was conducted by mixing with polyethylene glycol (PEG) as a carrier. PEG was provided by making a mixture of PEG-400 liquid and PEG-4000 crystal, with an 80% (20 g):20% (5 g) ratio. 18

The bone graft technology commonly inserts the graft powder or solution in fluid or water. One of the drawbacks is after inserting the graft, this material would get diluted or adrift and go out from the dental socket. PEG is a high molecular weight material or gel that can settle in liquid material. PEG is also known as a nontoxic material. 19 This study uses PEG as a bone graft carrier in inserting the alveolar socket.

*HAp* -BTG was weighted at 0.5 g, mixed into 24.5 g of PEG, and was mixed homogenously. Every rat was injected 0.1 mL of *HAp* -BTG-PEG (for the treatment group) or PEG (for the control group) into the dental socket.

Fifty Wistar rats were randomly selected into two groups (control and treatment with 25 each). Every 25 rats in the control groups were randomly chosen to the subgroup 3, 7, 14, 21, and 28 days to get five rats in every subgroup. The same method was used for the treatment group and every subgroup was kept in separate cage.

Anesthesia was administered using a combination of xylazine and ketamine with a ratio of 1:1 intramuscular injection. Termination was conducted with an anesthetic injection in the right posterior femoral region. Lower left incisor teeth extraction was done using incisor extraction forceps. The apical site of the postextraction socket was filled with PEG in group I (control) and PEG + *HAp* -BTG in group II (treatment), as much as 0.1 mL using a syringe. Furthermore, the extraction wound is sutured with simple interrupted sutures using 3–0 nonabsorbable black silk sutures. The rats were put in supine position for 4 hours to maintain *HAp* -BTG stay in the postextraction slot. The rats were evaluated on 3, 7, 14, 21, and 28 days.

On the 3rd, 7th, 14th, 21st, and 28th day, each of the 5 Wistar rats was terminated from each group, retracted, necropsied, decapitated, took a left mandibular bone fragment, and then immersed in 10% formalin solution for tissue fixation. After fixation, excision, and decalcification, the left mandibular jaw was processed for immersion in paraffin. Sections were made in a semiserial longitudinal manner with a thickness of 4 μm from the hemimandibular containing the alveolar socket at 60 µm intervals and examined by hematoxylin-eosin (HE) staining and immunohistochemical (IHC) examination.

HE staining was used to count the number of OBs and OCs. 16 IHC staining was used for examining BMP-2, OCN, OSX, and RUNX2 assay. 1 16 20 21

For the examination of BMP-2, OSX, OCN, and RUNX2 expressions, OB, and OC, we used a 1,000× magnification light microscope in 20 microscopic fields. The mean results per microscopic field were tabulated and analyzed with SPSS version 25.

## Results

The experiment was conducted from January 18, 2020 to March 7, 2020. There were 50 Wistar rats in 2 groups, 25 control and 25 treatment rats, spread into 3, 7, 14, 21, and 28 days subgroup. All 50 rats were alive until the end of the experiment.

Six preparations of evaluated variables for each rat's dental alveolar tissue were prepared and stained for each variable. The enumeration of cells per microscopic field, among 20 microscopic fields of 1,000× magnification, is shown in Table 1 .

**Table 1:** The number of cells per light microscopic field with 1,000 times magnification, on the histopathological examination (immunohistochemistry for BMP-2, RUNX2, OSX, OCN, and hematoxylin-eosin staining for OB, OC) of alveolar bone socket tissue

Days	Variables	Cell number (mean) ± SD		*p*
		Control group	Treatment group	
D-3	BMP-2	6.41 ± 1.83	9.83 ± 0.68	0.011
	RUNX2	5.04 ± 0.54	9.71 ± 0.86	0.000
	OSX	6.83 ± 2.33	8.53 ± 2.48	0.530
	OCN	7.59 ± 1.92	11.86 ± 1.45	0.004
	OB	6.21 ± 4.40	15.06 ± 0.97	0.009
	OC	10.95 ± 1.90	8.40 ± 0.60	0.021
D-7	BMP-2	10.76 ± 1.97	15.19 ± 1.61	0.009
	RUNX2	8.38 ± 2.56	12.20 ± 2.71	0.051
	OSX	6.93 ± 2.47	13.64 ± 1.49	0.001
	OCN	11.14 ± 2.02	13.20 ± 1.87	0.133
	OB	7.47 ± 2.62	11.89 ± 2.26	0.021
	OC	12.85 ± 2.31	4.50 ± 2.16	0.009
D-14	BMP-2	11.58 ± 2.65	17.11 ± 1.98	0.006
	RUNX2	9.51 ± 1.60	16.68 ± 3.19	0.004
	OSX	8.63 ± 2.83	14.38 ± 1.37	0.004
	OCN	10.13 ± 3.77	17.51 ± 1.87	0.004
	OB	8.00 ± 2.09	13.85 ± 1.83	0.002
	OC	12.05 ± 2.11	4.50 ± 0.73	0.001
D-21	BMP-2	10.67 ± 1.20	18.44 ± 1.05	0.000
	RUNX2	10.41 ± 1.79	17.59 ± 2.07	0.000
	OSX	10.38 ± 3.76	15.87 ± 3.40	0.041
	OCN	12.33 ± 2.17	17.04 ± 1.70	0.005
	OB	12.36 ± 3.51	17.33 ± 2.29	0.029
	OC	12.57 ± 2.22	3.67 ± 0.69	0.000
D-28	BMP-2	13.41 ± 2.00	17.16 ± 1.44	0.009
	RUNX2	12.90 ± 1.83	16.54 ± 2.21	0.022
	OSX	12.60 ± 1.83	16.44 ± 2.52	0.025
	OCN	13.42 ± 1.26	15.99 ± 1.23	0.055
	OB	14.68 ± 3.24	18.34 ± 1.98	0.047
	OC	15.18 ± 1.43	3.98 ± 0.73	0.000

Abbreviations: BMP-2, bone morphogenic protein-2; OB, osteoblast; OC, osteoclast; OCN, osteocalcin); OSX, osterix; RUNX2, runt-related transcription factor-2; SD, standard deviation.

**Fig. 1:**
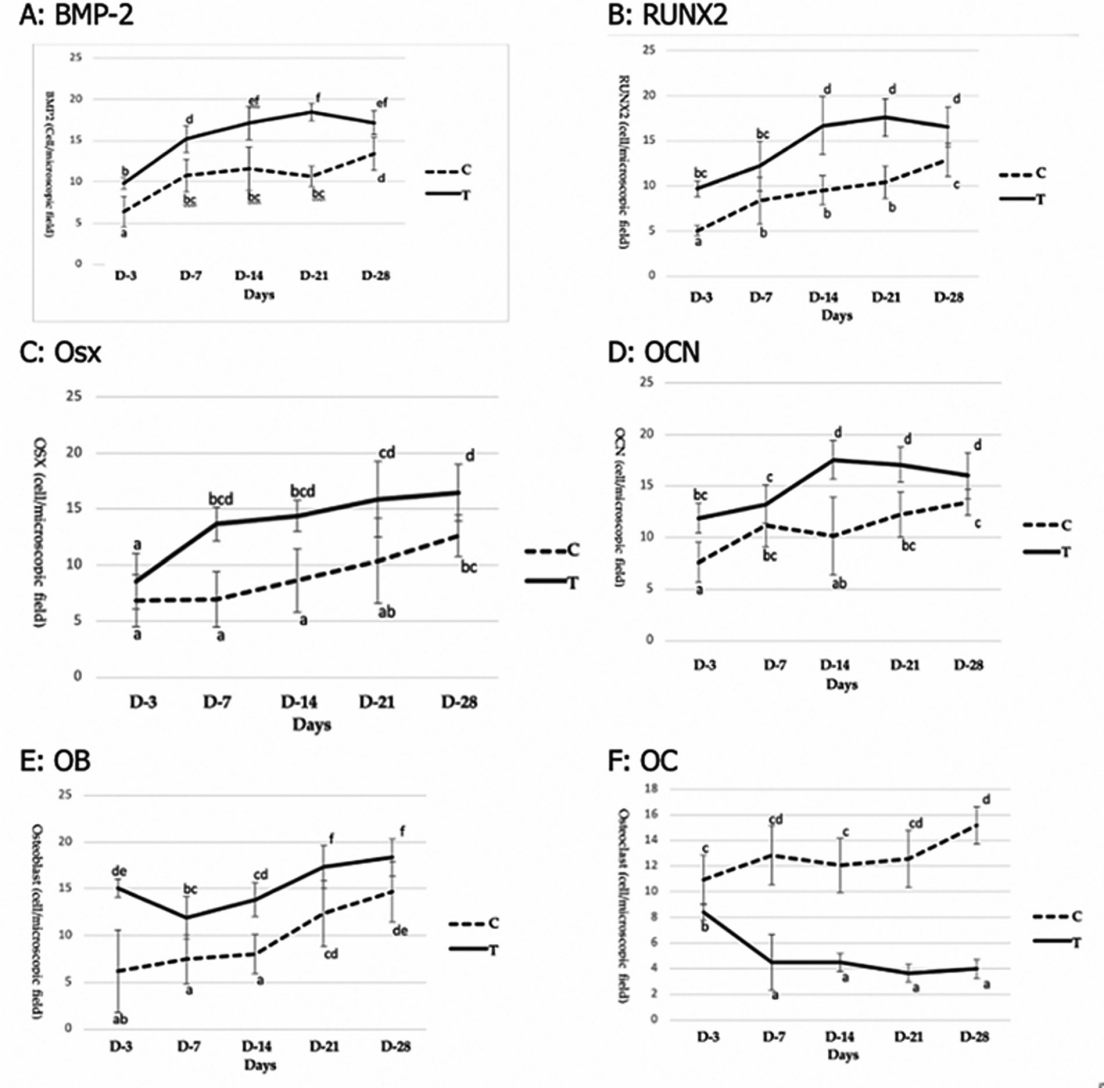
Comparison of mean of biomarkers expression between study groups and time/day of observation. Note: Different letter notations (a, b, c, d, e, f) indicate significant differences between groups; ( **A** ) bone morphogenic protein (BMP)-2, ( **B** ) runt-related transcription factor 2 (RUNX2), ( **C** ) osterix (OSX), ( **D** ) osteocalcin (OCN), ( **E** ) osteoblast, ( **F** ) osteoclast.

**Fig. 2:**
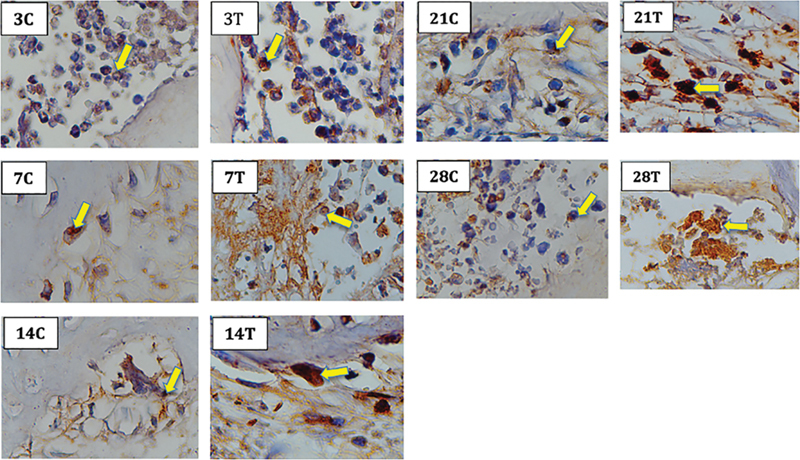
The results of immunohistochemistry (IHC) bone morphogenic protein (BMP)-2 examination on the alveolar bone socket of the Wistar rat's teeth showed a picture of osteoblast cells with BMP-2 expression marked in brown. Note: Numbers 3, 7, 14, 21, 28 = days of observation; C = control; T = treatment; yellow arrows = brown color are cells with BMP-2 expression.

The results of examining the number of cells expressing BMP-2 between the control and treatment group of observation days are given in Table 2 . The number of cells per microscopic field in the treatment group, days 3, 7, 14, 21, and 28, are higher and significantly different than the control. The increase in the number of cells in the treatment group occurred on days 3 to 7, 7 to 21, and then there was no significant change until day 28 ( Fig. 1 ). It shows the peak of BMP-2 expression was on day 21. Whereas in the control group, the number of cells increased significantly on day 7, there were no significant changes until day 28. The results of BMP-2 expression are shown in Fig. 1A . Cells those express BMP-2 are marked in brown ( Fig. 2 ).

**Table 2:** Description of mean, standard deviation, and difference test between groups of BMP-2 expression in the control and treatment groups on days 3, 7, 14, 21, and 28

Groups	Days of evaluation					*p*
	D-3	D-7	D-14	D-21	D-28	
Control	6.41 ± 1.83	10.76 ± 1.97	11.58 ± 2.65	10.67 ± 1.20	13.41 ± 2.00	< 0.001
Treatment	9.83 ± 0.68	15.19 ± 1.61	17.11 ± 1.98	18.44 ± 1.05	17.16 ± 1.44	< 0.01
*p*	< 0.05	< 0.01	< 0.01	< 0.001	< 0.01	

Abbreviations: BMP-2, bone morphogenic protein-2; D, days.

The results were similar for RUNX2 ( Table 3 , Fig. 1B ), OSX ( Table 4 , Fig. 1C ), and OCN ( Table 5 , Fig. 1D ). The number of cells expressing RUNX2 in the treatment group was higher and significantly different than the control group, except on day 7. RUNX2 in the treatment group showed an increase in expression from day 3 to 14, and there was no longer any significant difference until day 21 and 28.

**Table 3:** Description of mean, standard deviation, and difference test between RUNX2 expression in the control and treatment groups, on days 3, 7, 14, 21 and 28

Group	Evaluation days				*p*	
	D-3	D-7	D-14	D-21	D-28	
Control	5.04 ± 0.54	8.38 ± 2.56	9.51 ± 1.60	10.41 ± 1.79	12.90 ± 1.83	< 0.001
Treatment	9.71 ± 0.86	12.20 ± 2.71	16.68 ± 3.19	17.59 ± 2.07	16.54 ± 2.21	< 0.001
*p*	< 0.001	> 0.05	< 0.01	< 0.001	< 0.05	

Abbreviations: D, days; RUNX2, runt-related transcription factor-2.

**Table 4:** Description of mean, standard deviation, and difference test between groups of OSX expression in the control and treatment groups on days 3, 7, 14, 21 and 28

Groups	Evaluation days					*p*
	D-3	D-7	D-14	D-21	D-28	
Control	6.83 ± 2.33	6.93 ± 2.47	8.63 ± 2.83	10.38 ± 3.76	12.60 ± 1.83	< 0.05
Treatment	8.53 ± 2.48	13.64 ± 1.49	14.38 ± 1.37	15.87 ± 3.40	16.44 ± 2.52	< 0.01
*p*	> 0.05	< 0.01	< 0.01	< 0.05	< 0.05	

Abbreviations: D, days; OSX, osterix.

**Table 5:** Description of the mean, standard deviation, and difference test between groups of OCN expression in the control and treatment groups on days 3, 7, 14, 21 and 28

Groups	Evaluation days					*p*
	D-3	D-7	D-14	D-21	D-28	
Control	7.59 ± 1.92	11.14 ± 2.02	10.13 ± 3.77	12.23 ± 2.17	13.42 ± 1.26	< 0.05
Treatment	11.86 ± 1.45	13.20 ± 1.87	17.51 ± 1.87	17.04 ± 1.70	15.99 ± 1.23	< 0.01
*p*	< 0.01	> 0.05	< 0.01	< 0.01	> 0.05	

Abbreviations: D, days; OCN, osteocalcin.

The number of cells with OSX expression in the treatment group on days 3, 7, 14, 21, and 28 was higher and significantly different than the control group ( *p* < 0.05). Based on the evaluation days, there were no significant increase on day 3 compared with the control group, but it increased significantly on day 7. There was not a significant increase until days 14, 21 and 28. At the same time, in the control group, a significant rise in OSX expressing cells occurred on day 28 compared with day 3.

There was a significant increase in OCN expression from day 3 to 14, and then there was no significant increase until days 21 and 28 ( Fig. 1D ).

The examination of OBs with HE staining showed that since days 3, 7, 14, 21 and 28, the number of cells in the treatment group was significantly higher than the control group ( Table 6 ). While the cells counted between days of observation in the treatment group, the number of OB cells increased significantly on day 21 and then remained stable until 28 days ( Fig. 1E ).

**Table 6:** Description of mean, standard deviation, and difference test between groups of OB expression in the control and treatment groups on days 3, 7, 14, 21 and 28

Groups	Evaluation days					*p*
	D-3	D-7	D-14	D-21	D-28	
Control	6.21 ± 4.40	7.47 ± 2.62	8.00 ± 2.09	12.36 ± 3.51	14.68 ± 3.24	< 0.01
Treatment	15.06 ± 0.97	11.89 ± 2.26	13.85 ± 1.83	17.33 ± 2.29	18.34 ± 1.98	< 0.01
*p*	< 0.01	< 0.05	< 0.01	< 0.05	< 0.05	

Abbreviations: D, days; OB, osteoblast.

**Fig. 3:**
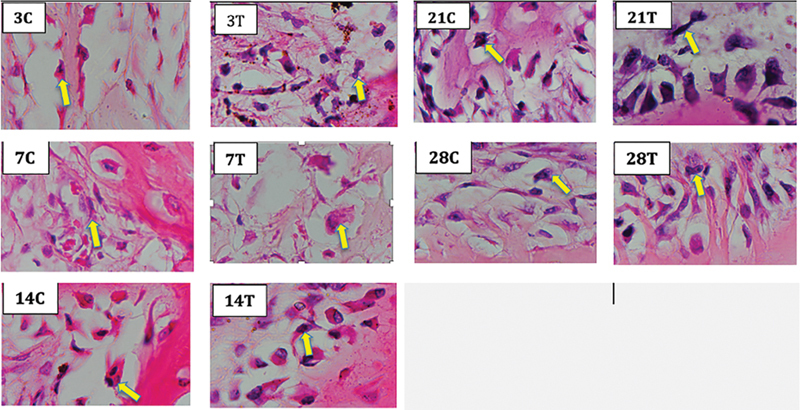
Osteoclast examination results in the alveolar bone socket of the *Norvegicus Wistar rat's* teeth, showing an image of osteoclast cells with multiple nuclear cells. Note: Numbers 3, 7, 14, 21, 28 = days of observation; C = control; T = treatment; yellow arrow = are osteoclast cells with multiple nuclei.

The results of the OC had opposite picture. Since days 3, 7, 14, 21, and 28, the number of cells in the treatment group was lower, and there was a significant difference compared with the control group ( *p* < 0.05) ( Table 7 ). While on evaluation days, a significant decrease occurred on day 3 to 7, then it remained constant, and there was no significant changes from day 7 to day 14, 21, and 28 ( Fig. 1F ). The results of OC cell expression are shown in Fig. 3 .

**Table 7:** Description of mean, standard deviation, and difference test between groups of OC expression in the control and treatment groups on days 3, 7, 14, 21 and 28

Groups	Evaluation days					*p*
	D-3	D-7	D-14	D-21	D-28	
Control	10.95 ± 1.90	12.85 ± 2.31	12.05 ± 2.11	12.57 ± 2.22	15.18 ± 1.43	< 0.05
Treatment	8.40 ± 0.60	4.50 ± 2.16	4.50 ± 0.73	3.67 ± 0.69	3.98 ± 0.73	< 0.01
*p*	< 0.05	< 0.01	< 0.01	< 0.001	< 0.001	

Abbreviations: D, days; OC, osteoclast.

## Discussion

*HAp* -BTG, as the test material in this study, is a particle resulting from calcination, sintering, and milling process, with a size of approximately 3.5 µm. In the treatment group, the increase in all osteogenesis indicators generally showed a significantly higher expression than the control group. It indicates the role of *HAp* -BTG in increasing the bone healing process in the area of the tooth alveolar socket.

It shows the peak expression in the treatment group was reached within 14 days (BMP-2, RUNX2, and OCN), and on day 7 (OSX). Meanwhile, the control group reached the peak on day 28 (BMP-2, RUNX2, OSX, OCN). The peak expression of the control group on day 28 (BMP-2, RUNX2, and OSX) was the same as that of the treatment group on day 7. It indicates that the *HAp* -BTG graft enhances and accelerates the process of osteogenesis. According to Thahir et al, the process of bone resorption and formation in male marmots takes approximately 2 to 4 weeks. 22

In the control group, OSX expression increased after day 3, but a significant increase was identified on day 28. In contrast, in the treatment group, a significant increase was identified since day 7 ( Fig. 1C ) and continued to increase until day 28. Also, the expression of OCN in the control group increased on day 7, and then there was no significant increase until day 28 ( Fig. 1D ); OB expression increased in the control group on day 28, but there was no significant difference with the treatment group on day 21 ( Fig. 1E ). From early to late healing stages, the mean expression of OBs was consistently increasing. Kamadjaja et al showed that the mean number of OBs was consistently higher in the treatment group by applying demineralized freeze-dried bovine bone xenograft than the control group in 2 to 4 weeks. 16

There was no significant change in the number of OC in the control group between day 3 to 7, day 7 to 14, day 14 to 21, and day 21 to 28. While in the treatment group, it decreased significantly on day 7, and then there were no significant changes from day 7 to 14, day 21, and day 28. It showed that OC has a pivotal role in the finalization process of alveolar tissue growth.

*HAp* scaffolds are *HAp* with a porous matrix where the size of the pores in *HAp* scaffolds can vary, depending on the volume of scaffold produced. It makes *HAp* scaffolds easier to implant into bone tissue, does not inhibit the growth of natural bone tissue, and can prevent displacement and loss of implants induced into the body. *HAp* scaffolds can serve as various materials, including polymers, ceramics, metals, and other composite materials. 23

Bovine tooth graft has a role in osteogenesis in alveolar bone defects. After inserting the bone graft to the alveolar bone defect, there will be a blob of bone graft wrapped in blood in the early stages. Then on the 7th day, there will be an acute inflammatory response with an invasion of neutrophil cells, lymphocytes, and plasma cells. The inflammatory process that occurs causes the activation of premesenchymal cells, growth factors, and inflammatory mediators that can cause premesenchymal cells to differentiate into OBs so that bone formation or osteogenesis will occur. 24

BMP-2 is well known to be a strong inducer of bone formation and to play important roles in the development and regeneration of bone and cartilage. 25 The expression of BMP-2 in the mechanism of osteogenesis in various groups can be seen in Table 2 and Fig. 1A , indicating significant differences between the treatment groups. The expression of BMP-2 shows a significant difference in the expression of BMP-2 on days 3, 7, 14, 21, and 28 ( *p* < 0.05) between the treatment groups compared with the control group. The administration of *HAp* -BTG in the treatment group showed an increase in BMP-2, significantly higher than the control group ( *p* < 0.001). BMP signaling is one of the central signaling pathways that induce osteogenic differentiation and regulate bone formation. BMP induces osteogenesis through the role of autocrine, paracrine hormones, and the action of RUNX2. 26

The osteoblastic differentiation and maturation events in the defect were evaluated by immunohistochemistry analysis which exhibits a significant increase in expressions of RUNX2. 16 RUNX2 expression in various groups are shown in Table 3 and Fig. 1B , which shows significant differences between the treatment groups and the control groups. Table 3 shows significant differences in RUNX2 expression on days 3, 7, 14, 21, and 28 ( *p* < 0.05) between the control and treatment groups. However, it significantly increased RUNX2 expression on days 3, 7, and 14. After that, it increased again on day 21. Then, there was no further increase until day 28 in the treatment group compared with the control group.

RUNX2 was first detected in pre-OBs and increased in the first week in immature OBs. At the fourth week, RUNX2 expression decreased during the maturation process of OBs, and RUNX2 expression was not significant in mature OBs. 27 It is consistent with the results of this study, which showed that RUNX2 expression increased on day 14 and then there was not any significant changes until day 28, and but were significant difference of RUNX2 on day 21 and 28 in the treatment group compared with the control group ( *p* < 0.05).

**Fig. 4:**
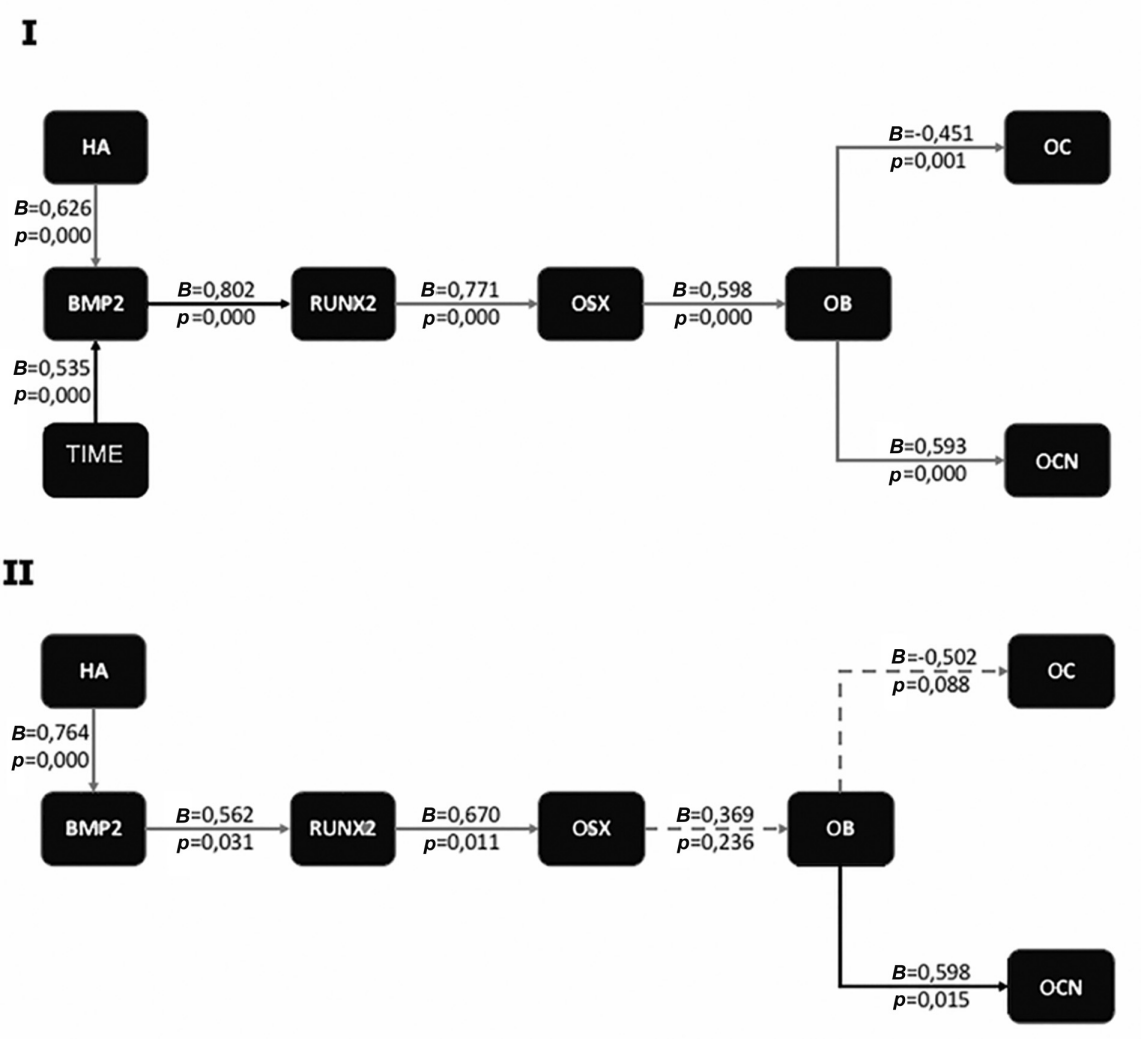
The mechanism of bone healing is based on the chain of interactions between biomarkers and the healing process of the dental socket after administration of hydroxyapatite bovine tooth graft ( *HAp* -BTG). Note: I = chart of treatment by time; II = chart on day 28; *p* = significancy, < 0.05 is significant; *B* = the magnitude of effect.

There was a significant difference between the treatment group and the control group. OSX expression on day 3 showed no significant increase in the treatment group compared with control ( *p* > 0.05). In contrast, on days 7, 14, 21, and 28, there was a significant difference in OSX expression in the treatment group compared with the control group ( *p* < 0.05) ( Fig. 4 ). OSX is a novel transcription factor of zinc finger, an essential element for OB differentiation and bone formation. 28 29 It plays an important role in the differentiation, maturation, or function of bone cells by regulating genes involved in different processes, and suggests a potential role in the bone microenvironment. 30

OB expression in the mechanism of osteogenesis in this study showed the number of OBs in various groups ( Table 6 and Fig. 1E ). On days 3, 7, 14, 21, and 28, the number of OBs in the treatment group was significantly higher than in the control group. ( *p* < 0.05). However, based on time observations, there was a significant decrease in OBs on day 7. On days 14 to 21, the number of OBs increased and continued to increase significantly until day 28. OBs are responsible for collagen production (type I collagen) and NCPs. It includes OCN, BSP, OPN, and osteonectin. OBs also express some ALP which helps mineralization. 31

On days 3, 7, 14, 21, and 28, the number of OCs in the treatment group was significantly higher than in the control group ( *p* < 0.05) ( Table 7 ). However, based on time observations, there was a significant decrease in the number of OCs on day 7, and then on days 14 and 21 to 28, the number of OCs remained low, and the same was observed as on day 7 ( Fig. 1F ). OCs are the major cells responsible for bone resorption. 16 OCs play a role in the bone resorption process. Hydrogen ions formed from carbonic anhydrase enter the plasma membrane to dissolve the bone matrix during the resorption process. Different lysosomal enzymes, namely collagenase and cathepsin K, are released to digest the bone matrix. 32 The low number of OCs indicates that bone growth continues throughout this experiment, which is indicated by high OBs and low OCs.

There is something new from this study: the mechanism of alveolar bone osteogenesis after administration of *HAp* -BTG was observed on the 3rd day until day 28 through the BMP-2, OSX, OCN, and RUNX2, OBs and OCs *in vivo* . The *HAp* -BTG scaffold material was proven to have the ability to induce osteogenesis in the alveolar bone socket of the *Rattus norvegicus strain Wistar rats* *in vivo* . This *HAp* -BTG scaffold has potential as a mineral because it secretes active metabolites such as cytokines and growth factors. These results show the pathway of the influence of *HAp* -BTG and BMP-2 expression. The expression of BMP-2 affects the expression of RUNX2, which is the beginning of the process of osteogenesis. RUNX2 plays a role in the differentiation of mesenchymal stem cells into osteoprogenitors. The RUNX2 pathway affects the expression of OSX, which is the final stage of the osteogenic process. The role of OSX is to induce the differentiation of osteoprogenitors into pre-OBs. The OSX pathway affects OCN expression, which indicates that pre-OBs differentiate into OBs. OCN is the most abundant protein matrix found in bone. OBs express OCN in the bone matrix during alveolar bone remodeling. 22

BMPs, as a transforming growth factor-b superfamily member, have a pivotal role in inducing bone healing process. The review by Salazar et al showed that the BMP superfamily affects almost all aspects of bone, cartilage, and joint biology. 33 Our study showed that the pathway analysis indicates the early role of BMP-2 in the chain process of socket bone healing after applying *HAp* -BTG ( Fig. 4I ). The expression of BMP-2 was impacted by *HAp* -BTG and by the time sequences. The analysis shows that BMP-2 was the first important starting point in bone healing after induction of *HAp* -BTG. The next biomarkers were RUNX2, OSX, OB, OCN, and OC. This picture shows that RUNX2, via OSX, induces OB differentiation from OB progenitor.

The previous study also showed the differentiation of OB via upregulating RUNX2 34 35 and OSX's role as an inducer. 30 36 The increase of OB is followed by increasing OCN. OCN may function as a matrix signal in the recruitment and differentiation of bone-resorbing cells. 37 This mechanism showed the role of OCN in the late stage of the bone healing process in cofunction OC.

Our study also showed that after 3weeks (day 21), there was no influence of OSX on OB; at 28 days, there was no effect on suppression of OC ( Fig. 4II ) It shows that osteogenesis continues to bone formation and remodeling. The OC is essential for controlling the remodeling in bone formation. 38 OC do not simply resorb bone but participate in a fine adjusted system with the bone producing OBs to maintain and improve the structural strength of bone tissue. 39 The activity of OCN is needed for the following steps of bone healing toward complete osteogenesis, not discussed in this study. Clinically, serum OCN is a marker for bone formation and improves glucose metabolism, connecting to OCN and glucose metabolism. 40

*Limitation of this study* : (1) There were only six variables, BMP-2, RUNX2, OSX, OCN, OB, and OC. Thus, the mechanisms in more detail need to be further explored with more biomarkers. (2) This study was conducted in rats, to show the model of the mechanisms of bone healing. To explore the impact of bone synthesis completely and remodeling, further study in large animal is required.

## Conclusion

(1) Postdental extraction, *HAp* -BTG administration in the dental socket can induce biomarker (BMP-2, RUNX2, OSX, OCN, OB) expression higher and faster, improving the process of bone healing.
(2) BMP-2 is the dominant biomarker first induced during the bone healing process, then RUNX2, OSX, OB, and OCN/OC.
(3) On the 28th day, it did not significantly express the suppression of OC by OB, which entered the bone formation and remodeling step.
